# A bittersweet fate: detection of serotype switching in *Pseudomonas aeruginosa*

**DOI:** 10.1099/mgen.0.000919

**Published:** 2023-01-11

**Authors:** Mikkel Anbo, Lars Jelsbak

**Affiliations:** ^1^​ Department of Biotechnology and Biomedicine, Technical University of Denmark, DK-2800 Kgs Lyngby, Denmark

**Keywords:** Antibiotic resistance, HIRIC evolution, lipopolysaccharide, O antigen, *Pseudomonas aeruginosa*, serotype switch

## Abstract

High-risk clone types in *Pseudomonas aeruginosa* are problematic global multidrug-resistant clones. However, apart from their ability to resist antimicrobial treatment, not much is known about what sets these clones apart from the multitude of other clones. In high-risk clone ST111, it has previously been shown that replacement of the native serotype biosynthetic gene cluster (O4) by a different gene cluster (O12) by horizontal gene transfer and recombination may have contributed to the global success of this clone. However, the extent to which isolates undergo this type of serotype switching has not been adequately explored in *P. aeruginosa *. In the present study, a bioinformatics tool has been developed and utilized to provide a first estimate of serotype switching in groups of multidrug resistant (MDR) clinical isolates. The tool detects serotype switching by analysis of core-genome phylogeny and *in silico* serotype. Analysis of a national survey of MDR isolates found a prevalence of 3.9 % of serotype-switched isolates in high-risk clone types ST111, ST244 and ST253. A global survey of MDR isolates was additionally analysed, and it was found that 2.3 % of isolates had undergone a serotype switch. To further understand this process, we determined the exact boundaries of the horizontally transferred serotype O12 island. We found that the size of the serotype island correlates with the clone type of the receiving isolate and additionally we found intra-clone type variations in size and boundaries. This suggests multiple serotype switch events. Moreover, we found that the housekeeping gene *gyrA* is co-transferred with the O12 serotype island, which prompted us to analyse this allele for all serotype O12 isolates. We found that 95 % of ST111 O12 isolates had a resistant *gyrA* allele and 86 % of all O12 isolates had a resistant *gyrA* allele. The rates of resistant *gyrA* alleles in isolates with other prevalent serotypes are all lower. Together, these results show that the transfer and acquisition of serotype O12 in high-risk clone ST111 has happened multiple times and may be facilitated by multiple donors, which clearly suggests a strong selection pressure for this process. However, gyrA-mediated antibiotic resistance may not be the only evolutionary driver.

## Data Summary

Dataset A (153 XDR genomes): DOI: 10.1093/jac/dkz147; accession: PRJEB31047.

Dataset B (692 genomes): DOIs: 10.1128/AAC.03954–14, 10.1128/mBio.01796–15, 10.1038/35023079, 10.1371/journal.pone.0008842, 10.1128/MRA.00865–19, 10.1128/JCM.00349–16, 10.1101/gr.086082.108, 10.1186 /GB-2006-7-10-R90, 10.1371/JOURNAL.PONE.0007740; accessions: PRJNA297679, PRJNA264310, PRJNA294638, GCF_000017205.1, GCF_000006765.1, GCF_000014625.1, GCF_000568855.2, GCF_000026645.1.

**Impact Statement**

The increasing prevalence of nosocomial infections by drug-resistant *Pseudomonas aeruginosa* is frequently linked to a limited set of epidemic high-risk clones (HIRICs) that are globally disseminated across hospitals. HIRICs are associated with poor clinical outcomes and a diverse collection of horizontally acquired resistance genes. However, the genetic mechanisms that enable the emergence of these particular clone types as HIRICs are unknown. Determination of the mechanisms that drive the evolution of clones into HIRIC status is of high importance in order to impede the continuing spread and emergence of novel and current HIRICs.

Genetic variations in clusters that modulate the surface of the bacteria are potentially one such biomarker that could predict the emergence of new HIRICs. Numerous studies have investigated the role of the lipopolysaccharide in bacterial pathogenesis by the use of knockout mutants, and found it critical for establishment of infection. Replacement of the native serotype gene cluster has previously been shown to play an important role in relation to the emergence of HIRIC ST111. Therefore, we suggest that serotype variations may be equally important for clonal success and tools that can detect such events are necessary.

## Introduction

*Pseudomonas aeruginosa* is a Gram-negative opportunistic pathogen known to be one of the most frequent causes of nosocomial infections [1]. The relative success of this pathogen can be ascribed to many factors, especially its high intrinsic antibiotic resistance, its arsenal of virulence factors and its metabolic diversity [2–4]. It also has an astounding ability to develop resistance to nearly all antimicrobials available, either by adaptive mutations or acquisition of mobile genomic elements, leading to multidrug-resistant (MDR) or extensively drug-resistant (XDR) strains [5]. While there is high clonal diversity of *P. aeruginosa* isolates, it has become clear that certain clones are very successful on a global scale. These global clones have been denoted high-risk clones (HIRICs) due to their association with the MDR/XDR phenotypes and prevalence [2, 6]. Examples of HIRICs include (in order of descending prevalence) ST235, ST111, ST175 and ST244 [7].

One of the major virulence factors of *P. aeruginosa* is the lipopolysaccharide (LPS), which interacts directly with the extracellular environment [8]. The LPS is an integral component of the Gram-negative cell envelope. It is a glycolipid that consists of three domains: lipid A, core polysaccharide and O-antigen. The O-antigen plays a major role in surface attachment, virulence and resistance to host defences [9–11].

There are two types of O-antigens: the common polysaccharide antigen (CPA), which is a polymer of d-rhamnose, and the O-specific antigen (OSA). The O-antigens serve as targets for antimicrobials such as bacteriophages; bacteriocins, e.g. R-, S- and L-pyocins; and opsonizing antibodies [12–15]. The O-antigens of *P. aeruginosa* also serve a protective role during infections, such as protecting against phagocytosis and serum killing [13, 16]. Both O-antigens can simultaneously be expressed on the surface of *P. aeruginosa *, but in cystic fibrosis-adapted isolates it is commonly observed that the CPA becomes the dominant O-antigen [17]. It has been observed that mutations that prevent production of the OSA are selected for during adaptation to chronic infections [17].

The OSA is a heteropolymer that consists of repeat units of two–five sugars, depending on the serotype of the cell [18]. The composition of the repeat unit defines the serotype, which is used in serotyping to divide this bacterium into distinct groups. In *P. aeruginosa* the serotype cluster resides on a replacement island located at the same locus for most serotypes [19]. This means that the serotype of an isolate is mutually exclusive in most cases. There are 20 different serotypes, which are differentiated by 13 different OSA gene clusters and the presence of a gene called *wzy*_β_ [20–22]. The presence of gene *wzy*_β_ is used to determine whether an isolate belongs to serogroup O2 (presence of *wzy*_β_) or O5 (absence of *wzy*_β_), since these serotypes share the same OSA cluster. Detecting these clusters and the presence of the gene *wzy_β_* enables *in silico* serotyping of strains using whole-genome sequencing data [23].

Using this *in silico* approach, it was recently observed that the evolution of HIRIC ST111 involved switching from serotype O4 to O12, as well as acquisition of a fluoroquinolone-resistant gyrase allele through horizontal transfer [24]. In this serotype switching process, the native biosynthetic OSA gene cluster (O4) is replaced by a different OSA gene cluster (O12) by recombination. Whether similar serotype switching events take place in other HIRIC clone types is currently not known. On a broader scale, the extent to which serotype switching takes place in *P. aeruginosa* is also poorly understood. Several surveys of *P. aeruginosa* have previously reported multiple serotypes within sequence types and similar isolates [2, 25–31], suggesting that serotype switching may play a role in these clones.

**Fig. 1.**
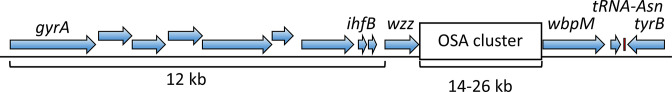
Schematic representation of the genes surrounding the O-specific antigen (OSA) cluster in *P. aeruginosa *. The OSA cluster varies in *P. aeruginosa* isolates and 11 different clusters have been described to reside in this locus [18], with the cluster sizes spanning 14–26 kb. Genes are represented by blue arrows, the OSA cluster locus by a white box, and the red box is the *tRNA-Asn*. The gene map is based on gene annotations in PA14.

This study presents a method that combines *in silico* serotyping and core-genome alignment using whole-genome sequences to detect serotype switches. We apply this method to two different datasets to evaluate the robustness of this method and estimate the prevalence of serotype switching in a broader clinical setting. We also examine the extent of recombination for serotype O12 isolates, which is possible due to the highly divergent serotype O12 donor strain(s), and the conservation of genes proximal to the serotype cluster, as illustrated in Fig. 1.

## Methods

### Isolates included in the study

*P. aeruginosa* genome assemblies were downloaded from the National Center for Biotechnology Information (NCBI) [32]. Many of the genomes are from different surveys of MDR/XDR clinical isolates of different geographical scope and size. Hence, the isolate genomes are divided into separate datasets, which are analysed separately. Dataset A is composed of 153 XDR isolates from a Spanish cohort study [25]. Dataset B is composed of 692 isolates derived from the bioMérieux strain collection [33], the Pirnay collection [34], IATS strains [23], the Kos collection [26], and reference strains PAO1 [35], PA7 [36], PAK [37], LESB58 [38] and PA14 [39].

### *In silico* serotyping

Serotype of isolates were determined using a blast [40] routine based on PAst [23] with some modifications. The OSA cluster database from PAst was updated to include recently discovered O15 and O17 clusters [22]. In addition, a new algorithm for matching fragmented queries to the database was incorporated. Practically, this is identical to the workflow of PAst when a query loci contains a complete OSA cluster. Any result with <95 % coverage to a serotype cluster is designated as non-typeable (NT). The non-typeable designation can either be due to deletions, mutations of the serotype cluster, or assembly quality issues. This was not further pursued.

If the query loci is fragmented, the new algorithm is used to assemble a complete OSA cluster using the blastn alignment. This algorithm attempts to find the best combination of non-overlapping (<5 % overlap cutoff) alignments from a given query to a specific OSA locus. The best combination is found by summing the bitscores of the resulting alignment combinations. This also gives a better approximation of coverage for serotype calling. Additionally, this enables reconstruction of OSA clusters from fragmented assemblies, which can be used for determining evolutionary relationships among serotype clusters.

### Sequence typing of isolates

Isolates were typed according to the multilocus sequence typing (MLST) scheme proposed by Curran *et al.* [41] using allele and sequence type (ST) designations from pubmlst.org [42]. A homemade R script based on blast alignments was then used to type isolates in this study, based on their genome sequence. For dataset A, sequence types were taken from a supplement of [25], since assembly quality was not sufficient for our script to assign STs.

### Core-genome alignment and phylogeny

Core-genome alignment and phylogeny was performed using ParSNP v1.5.4 [43] with flag ‘-c’. For datasets A and B, the reference genomes were set to accessions ‘GCA_900706705.1’ and ‘GCF_000006765.1’, respectively, using flag ‘-r’. This utilizes whole-genome sequences of assembled genomes.

### Detection of serotype switches

In order to detect serotype switching, we sought to design an algorithm that highlights cases where isolates are extremely similar, based on core-genome (as described in the previous paragraph), but have different serotypes. To do that we calculate *SND* based on branch distance from the tree, the number of serotypes and the number of isolates according to equation (1):

$$Equation1.\;SND=\frac{ICSR/n}{ICD}$$

**Fig. 2.**
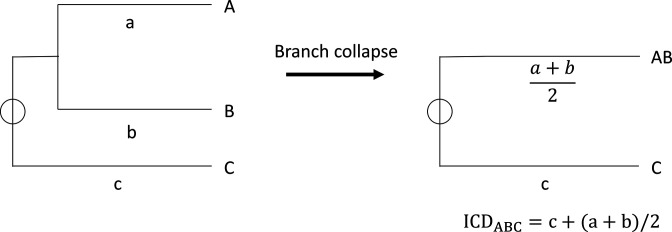
Calculating distances between isolates and clades by branch collapse. Conceptual view of how the distance measure *ICD* is calculated when a sub-branch is collapsed. The specific distances to isolates A, B and C are represented by lowercase a, b and c, respectively.

*ICSR* is the interclade serotype richness, which is the sum of different serotypes in a given clade. For this analysis, non-typeable isolates are not counted. *n* is the number of isolates in a given clade and *ICD* is the interclade distance, which is the summed distance of a given clade. The distance is found by iteratively collapsing sub-branches and finding the mean of the distances, as exemplified in Fig. 2. These parameters are calculated at all branching points of the tree (except the root) to find *SND*. A clade is defined as any branching point with >1 leaf that can contain either two leaves, a clade and a leaf, or two clades. A high *SND* value represents branches where serotype switches may have occurred.

*SND* is calculated for each branch of the tree and an *SND* threshold is necessary for more accurate predictions of serotype switches. We use the MLST of the isolates to set the threshold above the highest *SND* where >1 STs are involved. We have included the possibility to allow single-locus and double-locus variants for results with >1 ST, but for this study we have used the strictest threshold where no locus variants are allowed.

To count the number of serotype-switched isolates for a given ST, the majority serotype must first be determined for the ST. The number of serotype-switched isolates is the number of isolates, with the minority serotype, in the clade with an *SND* above the threshold. If there is no majority/minority serotype (i.e. *n*=2 isolates), only one isolate is assumed to have undergone a serotype switch. The direction of the serotype switch cannot readily be determined based on this analysis.

### Localization of PA7-like serotype genomic island

In order to determine if the O12 serotype cluster was obtained from a PA7 genomic island, all serotype O12 isolates were analysed using blast, in order to find highly similar alignments near the O-antigen cluster. This is done by blasting whole genomes against the genome of PA7 and subsequently stitching proximal alignments together using the following criteria: identity >=98 %, alignment length >100 bp, maximum gap length between different alignments, according to subject and query strand (where possible), must be <1000 bp.

### Gyrase subunit A alignment

The GyrA allele of all O12 isolates, eight representative ST111 O4 isolates;, and type strains PA7, PAO1, PA14, LESB58 and PAK were extracted using blast and aligned using muscle (v3.8.31) [44]. Gblocks (0.91b) [45] was used to eliminate any poorly aligned or highly divergent regions using the parameters ‘-t=d –b5=n’ and subsequently FastTree 2 (2.1.11–1) [46] was used to infer approximate maximum-likelihood phylogeny with parameters ‘-pseudo –spr 4 –mlacc 2 –slownni –nt’. Alleles were translated using NCBI genetic code ‘11’.

### Data visualization and analysis

Phylogenetic trees were visualized using ggtree [47]. Data analysis was performed using Rstudio (1.3.1093), R (4.0.3) [48] and visualized using ggplot2 [49]. Additional R packages used were Ragg, ggpubr, treeio, tidytree, viridis, ape, ggnewscale, seqinr, readr and rcolorbrewer [50–59].

## Results

### Serotype switching events are identified by the absence of linkage between O-antigen biosynthesis gene cluster and core-genome phylogeny

**Fig. 3.**
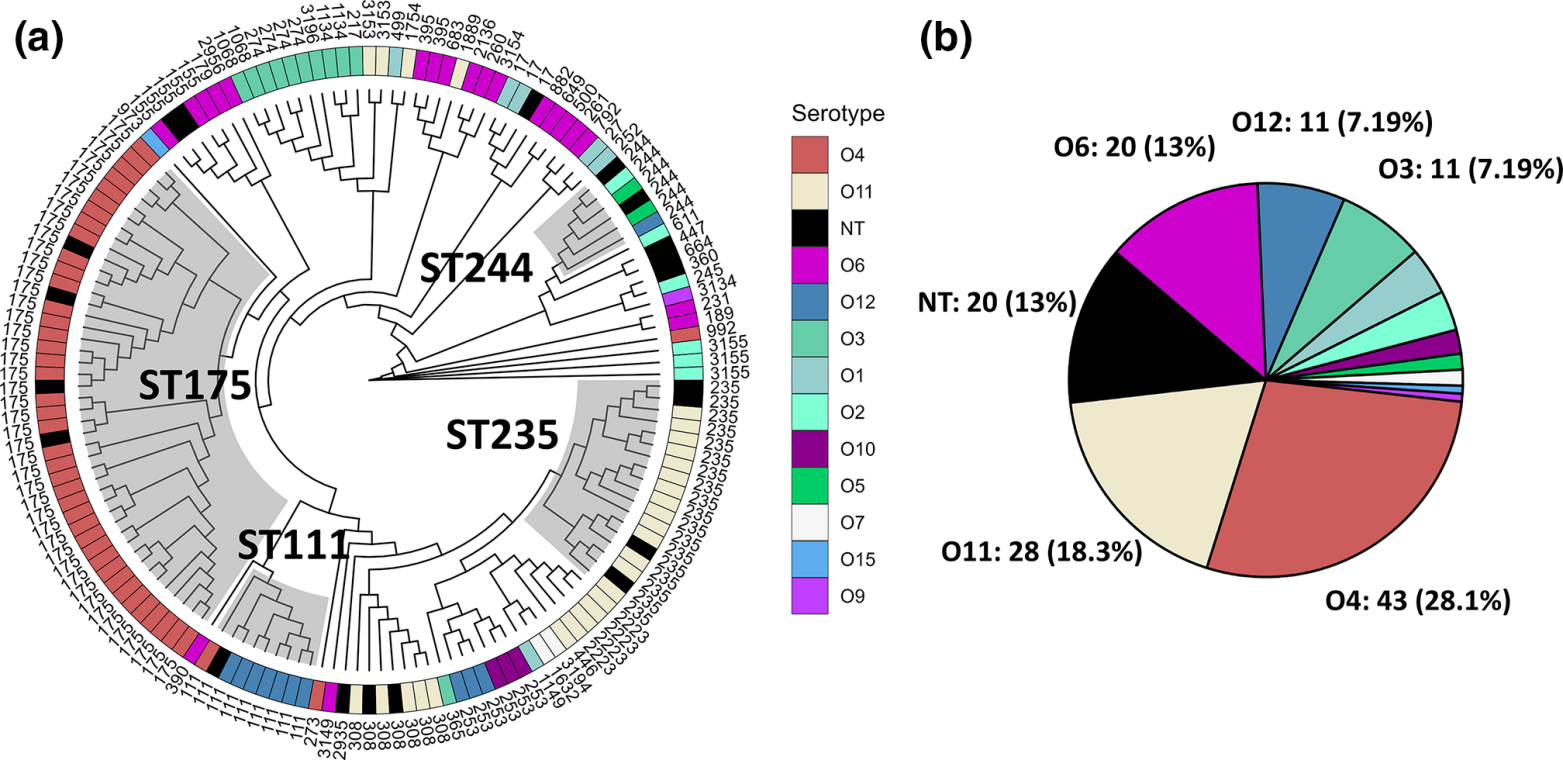
Phylogenetic relationship and serotype distribution of 153 XDR isolates. (a) Cladogram of the core-genome approximate maximum-likelihood phylogeny. On the cladogram, HIRICs ST244, ST235, ST111 and ST175 are highlighted with grey boxes. The tree has been annotated with the serotype (inner ring) and sequence type (ST) of the isolates (outer ring). The serotype colouring scheme is common to panels (a) and (b). The figure illustrates the linkage between serotype and core-genome phylogeny. (b) Serotype distribution of isolates in this dataset according to *in silico* serotyping. NT, non-typeable – isolates without a complete O-antigen cluster (<95 % coverage).

The aim of this study is to systematically identify and quantify cases where closely related isolates harbour different serotype clusters (an indication of a switch in serotype by acquisition and recombination of a new OSA cluster). In order to facilitate this analysis, a nationwide cohort study was analysed [25]. Whole-genome sequences of isolates (*n*=153) were *in silico* serotyped and core-genome alignment was performed using ParSNP [43] (1.5 MB core-genome). Approximate maximum-likelihood phylogeny was inferred with FastTree and visualized in Fig. 3a, where it can be observed that for the majority of clone types the serotype follows the sequence type (i.e. there is a linkage between core-genome phylogeny and serotype).

The distribution of serotypes in these isolates is shown in Fig. 3b. Serotype O4 isolates (*n*=43) and O11 isolates (*n*=28) account for almost half of the isolates due to the overrepresentation of HIRICs ST175 (O4: *n*=40) and ST235 (O11: *n*=14). Twenty isolates were found to be non-typeable (NT) due to the absence of a complete OSA cluster (isolates with <95 % coverage to a known OSA cluster). No serotype O13, O17, or multitypeable isolates were identified in this dataset.

However, in three cases, an absence of linkage between the OSA cluster and the core-genome phylogeny can be observed. ST244 isolates were found with three different serotypes: O2, O5 and O12. ST253 isolates were found with serotypes O10 and O12, and ST111 isolates were found with serotypes O4 and O12. We have previously observed similar examples of recombinational exchange of the O4 and O12 cluster in ST111 in other datasets [24], and we suggest that the cases in ST253 and ST244 are further examples of recombinational serotype switching events. This is substantiated by the fact that 97 % of ST253 genomes (*n*=189) downloaded from pubmlst.org belong to serotype O10. The same analysis of additional genomes from pubmlst.org of ST244 genomes (*n*=44) shows a varied distribution of serotypes, where 41 % belong to serotype O12, and the remaining isolates are evenly split between serotypes O2 and O5. Although ST244 has been reported to be associated with serotype O2 [7], our data suggest that serotype switching may contribute to the lack of a clear major serotype for this ST.

### Development of systematic approach to detect serotype switching

Using the previously described serotype switch cases found in the Spanish cohort study as a benchmark, we developed a systematic approach to detect serotype switches. Using phylogenetic distances as a measure of isolate similarity, we compute a parameter *SND* that highlights possible serotype switch cases. This parameter is calculated for each branching point of the phylogenetic tree where multiple (>=2) serotypes are present using phylogenetic distance, the number of serotypes and the number of isolates.

**Fig. 4.**
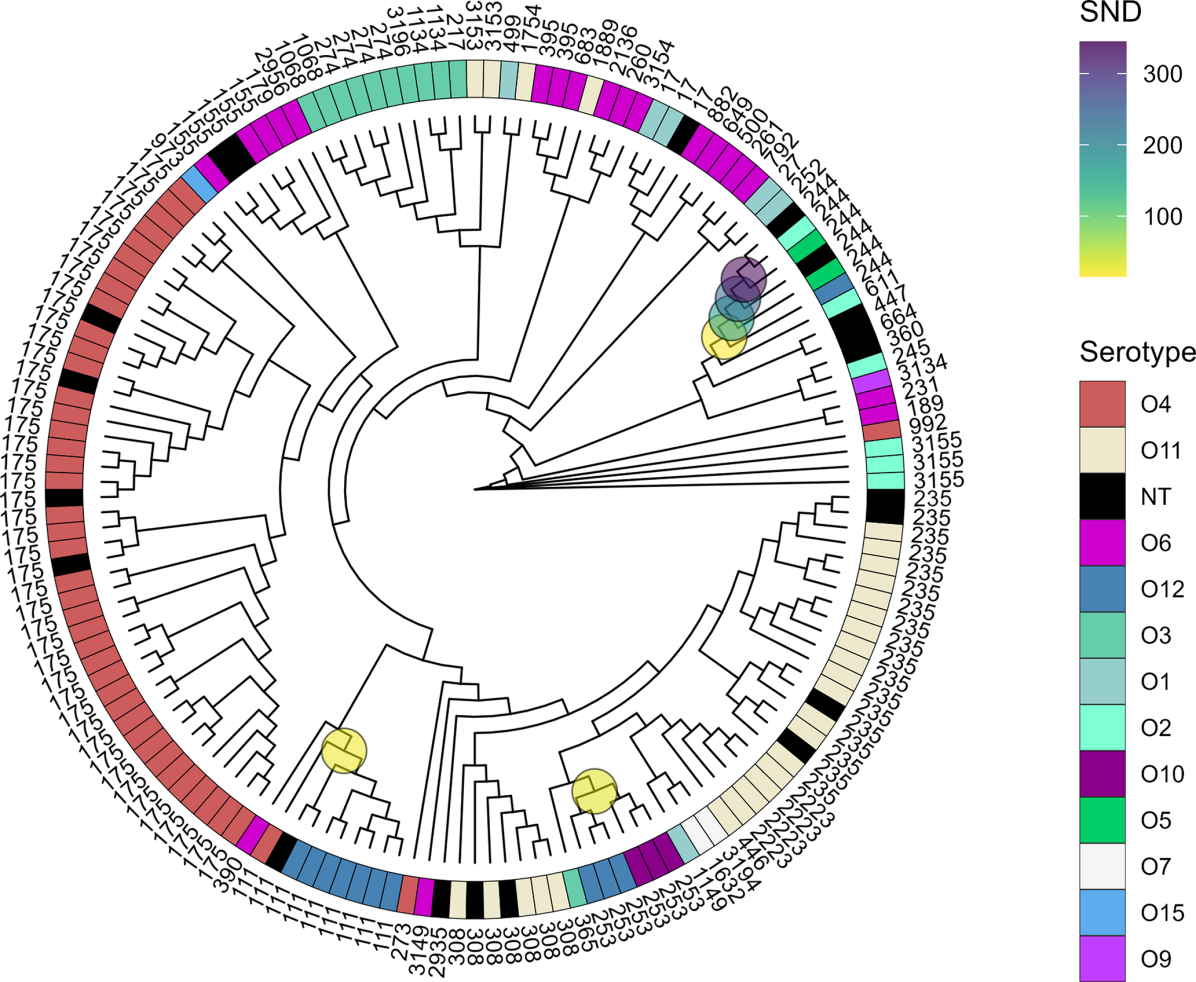
Systematic detection of serotype switching shown on cladogram of 153 XDR isolates. The cladogram shows serotype (inner ring) and sequence type (outer ring). Clades where serotype switches have been detected have been highlighted with circles. The circles have been coloured according to *SND*, which is highest in clades with highly similar isolates but differing serotypes.

This approach was applied to the aforementioned nationwide cohort study, and the result of the analysis was mapped onto the phylogenetic tree, as seen in Fig. 4. The analysis of this dataset shows serotype switching in the same three clades that were described in the previous section (Fig. 3). The number of serotype switches are quantified by counting the number of isolates with serotype(s) that differ from the majority serotype in a particular clade. In the case of ST111, which has one O4 isolate and seven O12 isolates, only one isolate would be counted as serotype switched. In total six serotype switches were detected, giving a prevalence of 6/153 = 3.9 % serotype-switched isolates.

The parameter *SND* is dependent on the size of the core-genome, since it uses phylogenetic distances, and it is used as an arbitrary measure of how likely a serotype switch is. The higher the *SND*, the more likely it is to reflect a true serotype switch, which means it is necessary to have a set of relatively closely related isolates in order to infer a serotype switch. Since the *SND* is computed for each branching point, a threshold *SND* has been imposed, which is described in the Methods section.

### Analysis of larger global dataset reveal serotype switching in HIRICs and non-HIRICs alike

Next, we sought to investigate whether this approach could be used to analyse a larger global survey of *P. aeruginosa* isolates (*n*=692). Isolates were *in silico* serotyped and core-genome aligned as previously described, with a resulting core-genome size of 230 kb. The three most prevalent serotypes for this dataset were (in order of descending prevalence) O11 (*n*=172, 25 %), O6 (*n*=137, 20 %) and O12 (*n*=68, 10 %). All serotypes have been represented in this dataset, and additionally, two multitypeable (MT) isolates were identified.

**Fig. 5.**
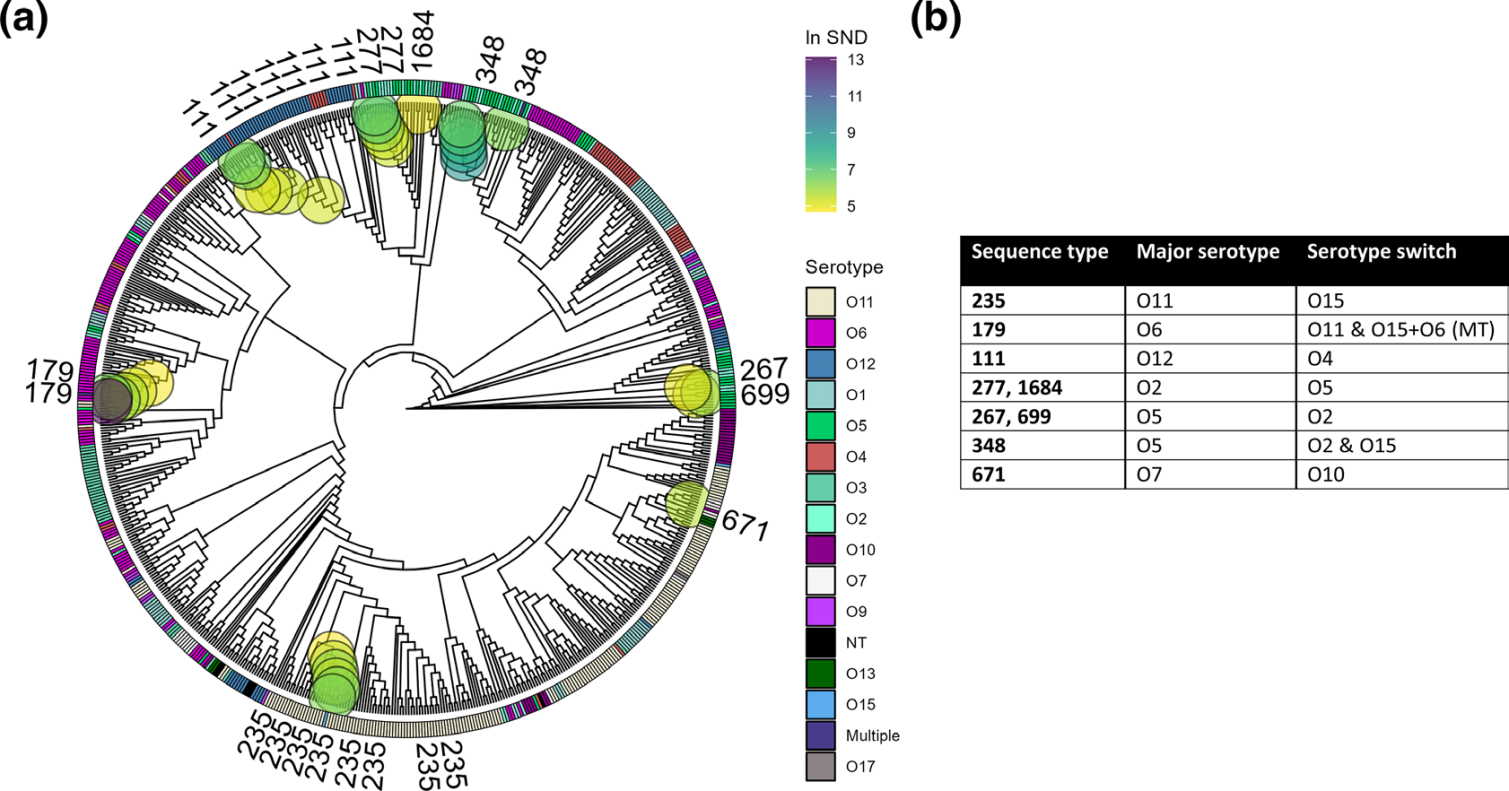
Serotype switch detection in a large global survey of *P. aeruginosa* isolates. (a) Approximate maximum-likelihood cladogram based on core-genome alignment of dataset B. The inner ring shows the serotype of the isolate. Serotype colour scheme is shown in the adjacent legend. The outer ring shows isolate sequence type. Only sequence types involved in serotype switches have been shown. Coloured circles within the tree indicate potential serotype switches as determined through our method. The colours of the circles indicate log *SND* score as shown in the adjacent legend. A high-resolution figure is available in Fig. S1 (available in the online version of this article), where all isolate STs and accessions are shown. (b) Table summarizing potential serotype switches. The table shows the relationship between sequence types and the major serotype found for a given ST, as well as the detected serotype switches. For ST235 we find that O11 is the major serotype, and a serotype switch to O15. For cases with multiple serotype switches as for ST179, we find that the major serotype is O6, and the serotype switches to either O11 or multitypeable (MT), where an isolate has both an O15 and an O6 cluster.

The core-genome alignment of isolates was analysed for serotype switches using the same methods as described previously. We identify 16 serotype switches in 9 different clades, resulting in a serotype switching prevalence of 16/692=2.3 %. The result of the analysis has been visualized on a cladogram, as seen in Fig. 5a, where isolates that are likely to have undergone serotype switching are highlighted with large coloured circles on the tree. In this dataset we find serotype switches in HIRICs ST235, ST111 and many other clone types (Fig. 5a). For ST235, we find a serotype switch between O11 and O15. For ST111, we find a serotype switch between O4 and O12.

Further inspection of the tree reveals the presence of two separate O4 clades within ST111 (Fig. 5a), but only the first clade is implicated in a serotype switch. The second O4 clade is more genetically divergent from the surrounding ST111 isolates and as a result does not meet the *SND* threshold. While this makes our method very conservative, it also makes the predictions more certain, since we limit ourselves to relatively recent evolutionary history (i.e. low genetic divergence). The same can be said for ST244 isolates, where we find three serotypes (O2, O5 and O12), but there is too much genetic diversity between these clades to infer a serotype switch.

There are also examples of multiple different serotype switches within certain clone types, such as for ST179. The majority of ST179 isolates are serotype O6, but we found an isolate with serotype O11 and a multitypeable isolate with both O15 and O6. The findings have been summarized in Fig. 5b, where for the aforementioned ST179 example the serotype switch is designated O11 and O15 +O6 (MT).

Overall, for this dataset we find a similar prevalence of serotype switching compared to the first dataset. Additionally, we detect a serotype switch in HIRIC ST111 in both datasets.

### Serotype O12 is obtained from a PA7-like serotype island

Our previous findings [24] suggested that the serotype O12 cluster originates from a PA7-like isolate, which belongs to a highly divergent group of *P. aeruginosa* isolates [60]. PA7 only shares approximately 93 % identity with other notable *P. aeruginosa* strains, whereas most other strains share upwards of 98–99 % identity [36]. This difference in sequence identity enables us to determine how much DNA is co-transferred with the serotype O12 cluster to non-PA7-like strains. This is done using high-identity whole-genome alignments of O12 isolates to PA7, using blast. These alignments are used to reconstruct and determine the extent of DNA transferred alongside the ‘serotype island’.

Since the assembly completeness of some of the O12 isolates is low, we may concatenate several alignments to infer the size and the boundaries of the serotype island. This is relying on the assumption that the location of genes near the serotype cluster is highly conserved. Additionally, since we may concatenate multiple contigs from fragmented assemblies, we have opted to report the boundaries of the serotype island(s) using PA7 genomic coordinates. A table of the coordinates can be found in Table S1. We have limited the analysis of the contents of the serotype island to *gyrA* in the following section.

**Fig. 6.**
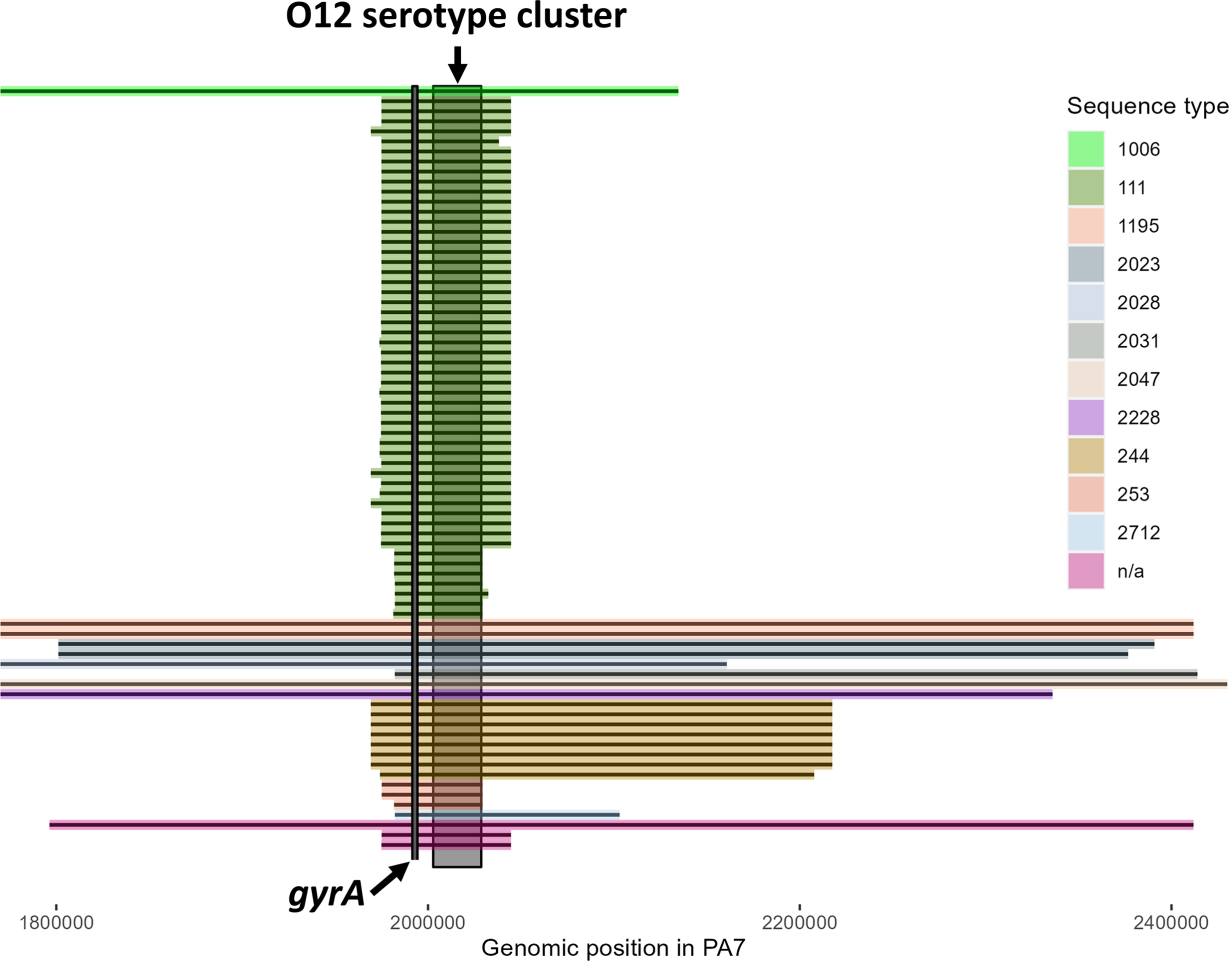
High-identity (>98 %) alignment of O12 isolates to the PA7 genome at the O12 serotype locus shows that the serotype island extends far beyond the serotype cluster. Additionally, the serotype island size is heterogeneous in size and boundary. Each alignment has been coloured by its sequence type and the location of the O-antigen cluster of PA7 has been highlighted with a dark box at the 2 MB mark. The location of *gyrA* has been marked with a black line on the figure. The coordinates on the axis refers to the genomic position in the PA7 genome. Isolates that could not be sequence typed are designated na due to incomplete or novel alleles.

Based on this analysis of 76 O12 isolates we found, in agreement with our previous findings [24], that recombinational exchange of the OSA cluster often extend far beyond the boundaries of the serotype gene cluster (Fig. 6). At the overall level, we found that that the size and boundaries of the serotype island (containing the OSA cluster and flanking DNA) correlates with the ST (for size: *P*<2.2e-16, ANOVA), and that the *gyrA* allele is co-transferred in every O12 isolate. For example, ST111 isolates have obtained serotype islands ranging from 47 to 70 kb and ST244 isolates have obtained serotype islands of ~250 kb (Fig. 6). However, at the clone type (ST) level, we also find differences in the size of the serotype island. For example in ST111, all the Spanish isolates (*n*=7) have serotype islands that are <50 kb, while the remaining ST111 isolates (*n*=45) have serotype islands that are >69 kb (*P*≈1.7e-05, Wilcoxon rank sum test). A similar case can be found in ST244, where one Spanish isolate has a serotype island 15 kb smaller than all other ST244 isolates. This suggests that multiple independent serotype switch events have taken place and there is a certain selective pressure for the serotype switch and the maintenance of O12.

### DNA gyrase subunit A alignment suggests multiple serotype O12 donors

Due to the heterologous size and location of the O12 serotype islands, an alignment of the *gyrA* gene was made in order to classify these. This alignment is made using representative O12 ST111 isolates (from datasets A and B), O4 ST111 isolates, PA7-like isolates (STs: 1195, 2028, 2211, 1006, 2039, 2031, 2228, 2023, 2047), and type isolates PA7, PAO1, PA14, LESB58 and PAK, for reference.

**Fig. 7.**
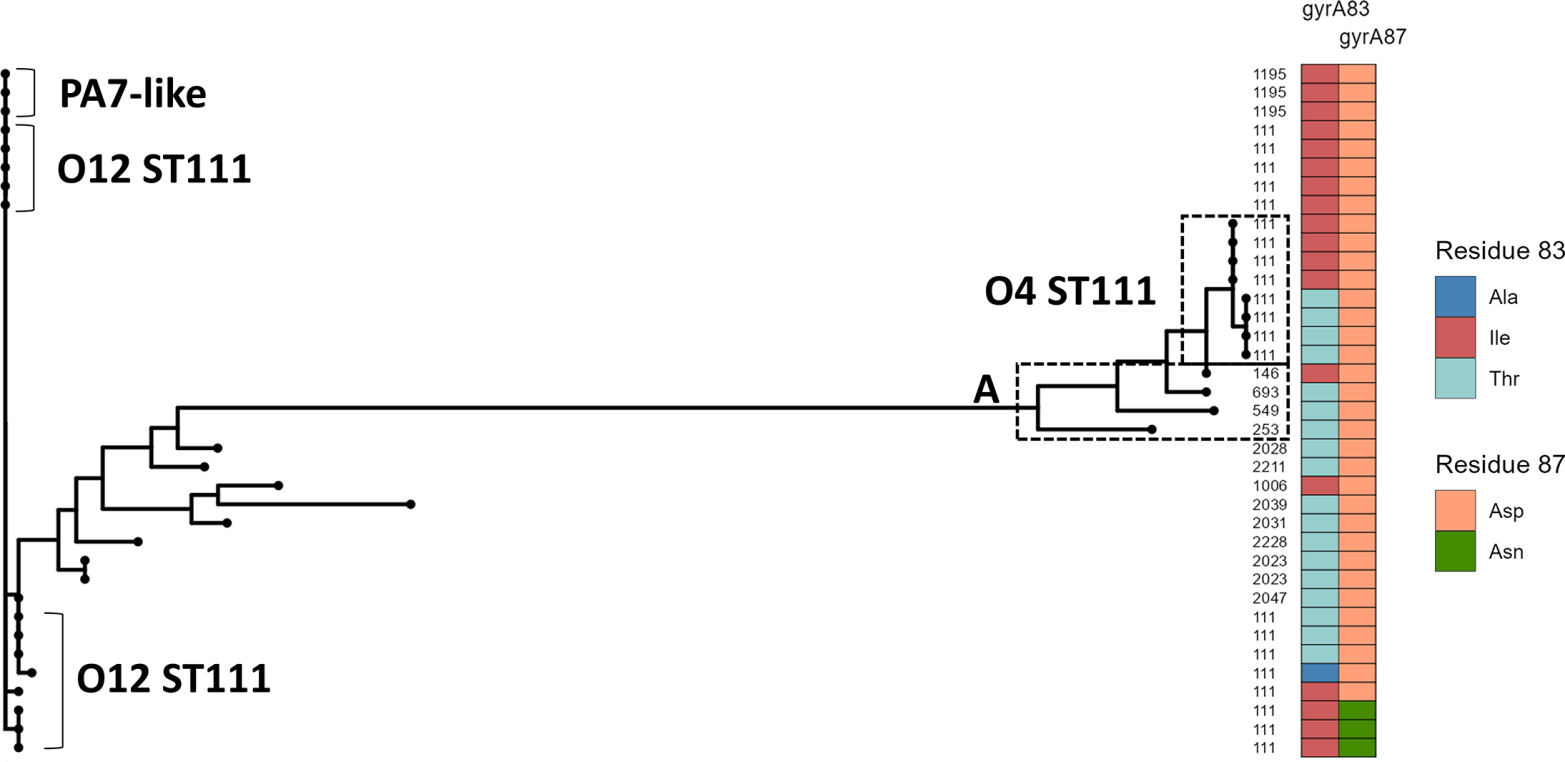
*gyrA* nucleotide alignment of ST111 isolates indicate multiple serotype O12 donors. Approximate maximum-likelihood tree annotated (from left to right) with sequence type, residue 83 and residue 87. ST111 isolates have resistant and susceptible *gyrA* sequences regardless of serotype. Type isolates (from top to bottom): LESB58, PAK, PAO1 and PA14.

Single amino acid substitutions in GyrA are known to confer increased resistance against fluoroquinolone antibiotics, particularly residues 83 and 87 [61, 62]. T83I, T83A and N87D mutations can be found in these previously mentioned isolates, which all have been previously shown to confer increased resistance to fluoroquinolones. The residues have been highlighted in the *gyrA* alignment shown in Fig. 7, where it is apparent that serotype O12 ST111 isolates cluster together with PA7-like isolates, and that O4 ST111 isolates cluster together with known type isolates such as LESB58.

However, what is also evident from this alignment is that resistance mutations are not absolute for either group of ST111 isolates, as both groups have susceptible and resistant alleles. We interpret this as an indication of multiple serotype O12 donors and, additionally, that quinolone resistance may be secondary in terms of evolutionary pressure for the uptake and maintenance of serotype O12.

Based on this analysis we find that 95 % of O12 ST111 isolates (*n*=52) have a resistant *gyrA* allele, while this is only the case for 50 % of O4 ST111 isolates (*n*=8). Extending this analysis to all O12 isolates from both datasets, we find that 86 % of isolates (*n*=79) have a resistant *gyrA* allele. For comparison, the proportion of resistant *gyrA* alleles in other isolates, categorized by serotype, is 62.5 % of O11 isolates (*n*=200), 28 % of O6 isolates (*n*=157) and 75 % of O4 isolates (*n*=92) (full list of results in Tables S2 and S3).

## Discussion

Much effort has gone into studying the bacterial surface and understanding the role of lipopolysaccharide (LPS) in relation to infection. However, the role of the 20 different variants of the O-antigen remains a seldom-studied subject in the context of the human pathogen *P. aeruginosa *. This study presents a method to detect serotype switching in genomic surveys of *P. aeruginosa *. Our results show that approximately 2–3 % of isolates were found to have undergone a serotype switch. The analysed datasets comprised antibiotic-resistant isolates, including high-risk clone types (HIRICs) such as ST111, ST175 and ST235. Additionally, our results show serotype switches in both HIRICs and non-HIRICs, suggesting that the serotype of a clone type may be more dynamic than previously thought.

We propose that serotype switches can be detected by the presence of very similar strains with different serotypes in *P. aeruginosa *, as has previously been shown for several other pathogens, such as in *Haemophilus influenzae* [63], *Streptococcus pneumonia* [64, 65], *Neisseria meningitis* [66, 67] and *Klebsiella pneumonia* [68, 69]. In order to infer similarity between strains, we have opted to use core-genome alignments. An argument could be made that multilocus sequence typing (MLST) is made for that exact purpose, but its strength lies in the grouping of strains on a higher taxonomic level using only seven housekeeping genes. Nonetheless, we employ MLST to set the threshold for the serotype switch detection limit. Using core-genome alignments, we are able to infer a conservative and high-resolution estimate of strain similarity. This also provides a more recent snapshot of intra-clone type evolutionary history.

The serotype O12 gene cluster seems to originate from a PA7-like strain, which is phylogenetically distant from most *P. aeruginosa* isolates [36]. This enables us to investigate the extent and boundaries of the DNA transferred during a serotype switch. From this analysis, it was found that serotype switching to O12 consistently involved the transfer of the *gyrA* allele. This gene is known for its role in fluoroquinolone resistance when certain residues are mutated. However, we find that O12 isolates do not exclusively harbour resistant *gyrA* alleles. While we do find differences in the proportion of resistant *gyrA* alleles between serotype O4 and O12 ST111 strains, there are too few isolates to make meaningful comparisons. Additionally, since the datasets that were analysed have specifically focused on MDR/XDR strains, any associations between resistance and other variables may be biased.

It was also found that the boundaries of the horizontally acquired DNA varied extensively, even for isolates of the same clone type, indicating different O12 donors, multiple serotype switch events, or different mechanisms of DNA transfer. An alternative explanation is that the recipients of the O12 serotype have different levels of genomic plasticity, which further complicates the interpretation.

Changes to the O-antigen structure were described previously by Holloway and Cooper, who relayed a change in the antigen of a bacteriophage D3 lysogenized strain [70]. Herein they found that the D3 lysogenized strain could no longer adsorb the same phage and that anti-sera prepared against the lysogenized strain and the non-lysogenized strain differed. From this they concluded that there must be a difference in the antigen structure of the two strains. This was further characterized by Kuzin and Kropinski, who elucidated the molecular details and showed that the D3 phage caused serotype conversion from O5 to O16 (serogroup O2) [71]. In a genomics study by Thrane *et al*., the authors concluded that the HIRIC ST111 must have undergone a serotype switch from O4 to O12 due to the presence of a serotype island with high homology to a PA7-like strain [24].

Multiple observational surveys of *P. aeruginosa* have reported the presence of isolates of the same clone type expressing different serotypes. Recio *et al*. reported the presence of an O1 ST235 isolate [27]. ST235 is usually associated with serotype O11. Abdouchakour *et al*. reported the presence of an O4 ST308 isolate, which is usually associated with O11 [28]. Furthermore, multiple previous studies have reported serotype O4 and O12 isolates of ST111 [2, 25, 26]. Miyoshi-Akiyama *et al*. reported multiple serotypes for STs 277, 244 and 357 [29]. Sekiguchi *et al*. reported strains with similar pulsed-field gel electrophoresis patterns but different serotypes [30]; moreover these were later typed as ST235 [31]. We suggest that most of these cases represent serotype-switched sub-populations, in which the native serotype cluster has been exchanged for another by homologous recombination. The presence of these serotype-switched populations may represent the emergence of a novel high-risk clone type, or the remainder of a less successful ancestral strain based on prevalence.

To our knowledge, this is the first attempt to determine the prevalence of serotype switching in *P. aeruginosa* isolates using a whole-genome phylogenetic approach. Most of the analysis can be conducted rapidly on a laptop with 8 GB RAM, with the exception being core-genome alignments for large datasets (approximately >150 genomes). The same method can potentially be used to study serotype switching in other pathogens, provided the biosynthetic pathways for serotype clusters have been elucidated.

For core-genome alignments, the core-genome size decreases as the number of genomes increases. The implication of this is that it becomes increasingly difficult to infer similarity between isolates. For this reason, an absolute cutoff for *SND*, in relation to serotype switch detection, cannot be made. In addition, *SND* values from datasets of different sizes cannot readily be compared due to core-genomes of varying sizes. In order to develop this algorithm further, researchers should aim to remedy these limitations by taking the size of the core-genome into account.

To understand the evolution of *P. aeruginosa* HIRICs, it is important to study the role of the O-antigen structure. In this paper, we present the first attempt to systematically detect serotype switches and we provide an approximate figure as to how often it happens in epidemic isolates of *P. aeruginosa *. We show that serotype switching is relevant to understanding the evolution of HIRICs, and that the serotype may be more dynamic than previously thought.

## Supplementary Data

Supplementary material 1

Supplementary material 2
