# Supplementary figures and images for: A bittersweet fate: detection of serotype switching in Pseudomonas aeruginosa
Source: Microb Genom. 2023 Jan 11;9(1):mgen000919. doi: 10.1099/mgen.0.000919 (PMC9973846; doi:10.1099/mgen.0.000919)

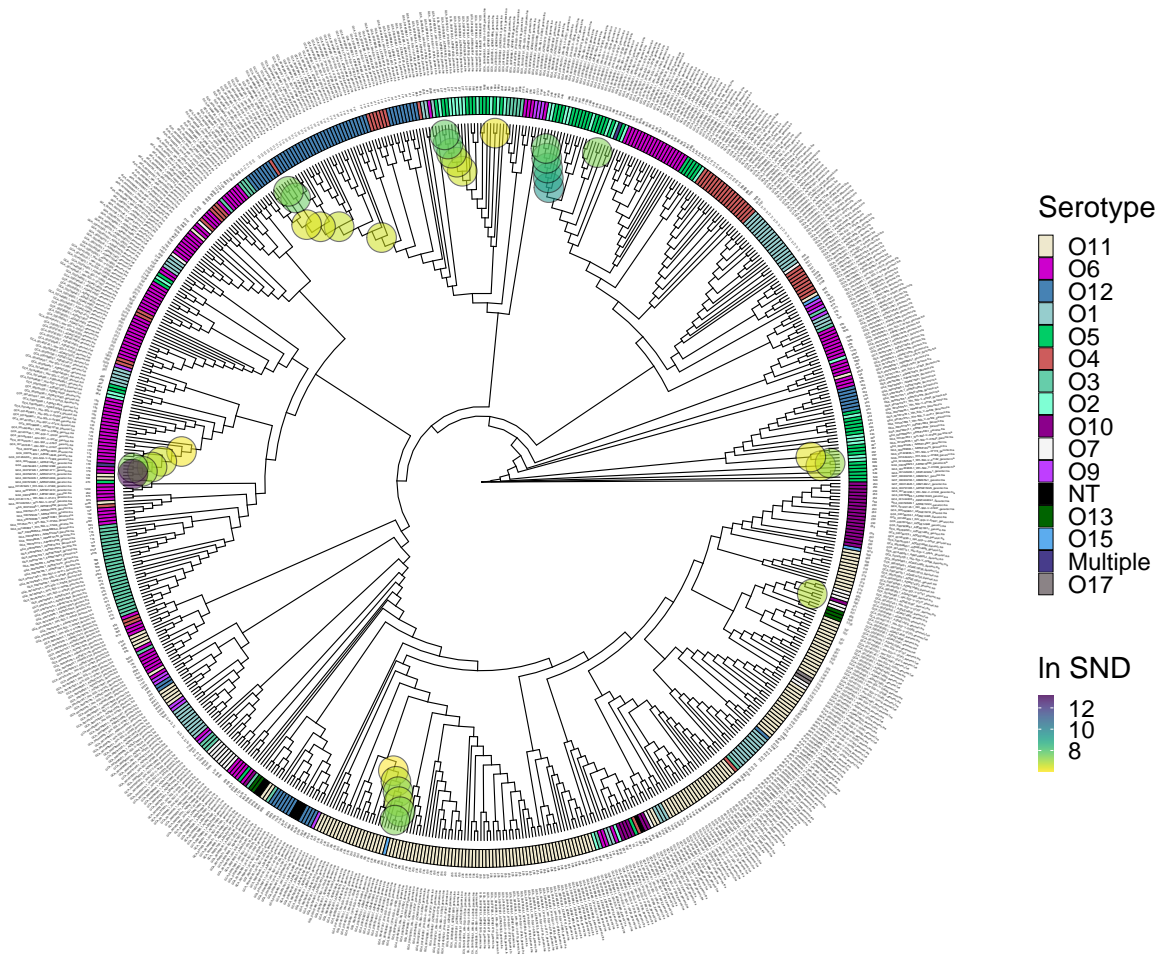

Supplement: Supplementary material 2 [file mgen-9-919-s001.pdf]
